# Entropy Generation in Peristaltic Transport of Hybrid Nanofluids with Thermal Conductivity Variations and Electromagnetic Effects

**DOI:** 10.3390/e25040659

**Published:** 2023-04-14

**Authors:** Abdulwahed Muaybid A. Alrashdi

**Affiliations:** 1 School of Computing and Mathematical Sciences, University of Leicester, Leicester LE1 7RH, UK; amaa20@leicester.ac.uk; Tel.: +44-7768-747752; 2 Mathematics Department, College of Science, Jouf University, Sakaka P.O. Box 2014, Saudi Arabia

**Keywords:** entropy generation, peristalsis, thermal radiation, ohmic heating, Homotopy Analysis Method

## Abstract

Entropy generation in peristaltic transport of hybrid nanofluid possessing temperature-dependent thermal conductivity through a two-dimensional vertical channel is studied in this paper. The hybrid nanofluid consists of multi-walled carbon nanotubes mixed with zinc oxide suspended in engine oil. Flow is affected by a uniform external magnetic field, hence generating Lorentz force, Hall and heating effects. Given the vertical orientation of the channel, the analysis accounts for mixed convection. To study heat transfer in the current flow configuration, the model considers phenomena such as viscous dissipation, heat generation or absorption, and thermal radiation. The mathematical modeling process employs the lubrication approach and Galilean transformation for enhanced accuracy. The slip condition for the velocity and convective conditions for the temperature are considered at the boundaries. The study analyzes entropy generation using the Homotopy Analysis Method (HAM) and includes convergence curves for HAM solutions. Results are presented using graphs and bar charts. The analysis shows that higher Brinkman and thermal radiation parameters result in higher temperatures, while higher thermal conductivity parameters lead to reduced entropy generation and temperature profile. Additionally, higher Hall parameter values decrease entropy generation, while an increased Hartman number improves entropy generation.

## 1. Introduction

Peristaltic type flows are significant due to their widespread applications in physiology and industry. Such flows find various applications in transporting sensitive and corrosive fluids, ranging from the movement of food, urine, and bile to roller and finger pumps in industrial processes. The broad utility of peristaltic flows has motivated researchers to analyze various aspects of such flows. Despite numerous investigations, there remain several unsolved issues, making further research in this field crucial. To contextualize this study, it is essential to discuss previous research and its limitations. For example, Hayat et al. [1] explored the movement of Carreau-Yasuda fluid via a curved channel as an application of flow in roller pumps. However, their study did not incorporate the effects of hybrid nanofluids, which possess unique chemical and mechanical properties that can significantly impact fluid flow and heat transfer. Similarly, Abbasi et al. [2] conducted an entropy generation analysis for the peristaltic flow of nanofluid and Ahmed [3] investigated the effects of mixed convection and Joule heating on the peristaltic flow of Sisko material, but these studies did not explore hybrid nanofluids, which offer superior mechanical and chemical properties. On the other hand, Rafiq et al. [4] also reported on the influence of Hall and Ion-Slip effects on the peristaltic flow of nanofluid. The use of nanomaterials in modern biotechnology and biomedicine has made the analysis of the peristaltic flow of nanofluids and hybrid nanofluids additionally important. Ready [5] investigated the biomedical aspects of blood flow considering peristalsis of hybrid nanofluid, incorporating the effects of thermal radiation and heat source/sink into the analysis. This study provides insights into the behavior of hybrid nanofluids under peristaltic flow, which can aid in developing more precise models for various processes. While previous research has made valuable contributions to the field, further investigations are required to develop a more comprehensive understanding of the peristaltic flow of hybrid nanofluids. Recent studies [6,7,8,9,10,11,12,13] have considered peristalsis of nanofluids/hybrid nanofluids, demonstrating the increasing interest in this area of research. Additionally, several investigations [14,15,16] have analyzed the entropy generation and flow characteristics of nanofluids, but few have considered hybrid nanofluids.

The use of nanomaterials in modern biotechnology and biomedicine has made the entropy generation analysis of the peristaltic flow of nanofluids and hybrid nanofluids increasingly important, see for instance [17,18,19]. Although previous studies have explored entropy generation analysis and the flow characteristics of nanofluids, these studies have not adequately addressed the effects of variable thermal conductivity and convective boundary conditions in the context of hybrid nanofluids in peristaltic flows. This is an important gap in the literature, given the widespread use of hybrid nanofluids in modern biotechnology and biomedicine. Therefore, this study aims to fill this gap by investigating entropy generation for the peristaltic flow of hybrid nanofluids consisting of Multi-Walled Carbon Nanotubes (MWCNT) and Zinc oxide (ZnO) suspended in engine oil. In particular, this study investigates the impact of mixed convection in a vertical symmetric channel, as well as the influence of an external magnetic field perpendicularly applied to the channel. The magnetic field generates a Lorentz force that affects the flow, and the study takes into account Hall and Ohmic heating effects caused by the magnetic field. The hybrid nanofluid is supposed to have temperature-dependent thermal conductivity, allowing for a comprehensive analysis of heat transfer. The study also considers other factors that could affect heat transfer, such as viscous dissipation, heat generation or absorption, and thermal radiation effects. To analyze temperature distribution, the study accounts for slip conditions for velocity and convective heat transfer at the boundary. To solve the resulting problem, the Homotopy Analysis Method (HAM) is utilized, and the study presents convergence curves for the solutions to aid readers. References [20,21] offer insights into fluid flows through analytical and numerical studies.

The uniqueness of this investigation lies in its combination of several complex phenomena, such as peristaltic transport, hybrid nanofluid, magnetic field effects, and mixed convection, and its comprehensive analysis of the flow behavior and entropy generation using advanced mathematical and computational methods. Specifically, the HAM is applied to the governing equations that describe the flow behavior and temperature distribution, which are highly nonlinear and coupled. The use of HAM allows for analysis of the entropy generation in the flow more accurately and efficiently than other methods. HAM can also provide insights into the convergence properties of the analytical solutions and can be used to generate convergence curves that show the accuracy and stability of the solutions. Additionally, the study considers various factors that can affect the heat transfer and temperature distribution, and provides insights into the relationships between these factors and the flow parameters.

This study aims to investigate the behavior of the hybrid nanoparticles under certain flow conditions, potentially with applications in nanofluidics or microfluidics. Specifically, the study considers the transport of an electrically conducting pattern of hybrid nanoparticles consisting of multi-walled carbon nanotubes mixed with zinc oxide suspended in engine oil. This transport occurs through a 2D incompressible peristaltic flow within a symmetric channel. The flow is driven by sinusoidal waves that propagate with a certain wavelength along the channel walls at a constant wave speed. The motion of the fluid is described using the equations of mass, momentum, and energy conservation, taking into account no-slip boundary conditions at the walls of the peristaltic channel. The non-dimensional process is conducted, and the solutions are presented using the HAM. The study further presents graphs for velocity, temperature, and entropy generation for variation in varied parameters to facilitate the physical analysis of the outcomes and to provide insights into how different factors affect the behavior of the system being studied. Bar charts are also employed to offer a comprehensive understanding of the heat transfer occurring at the walls across several values of parameters. The discussion section of the study presents a physical analysis of the results, and key outcomes are summarized at the end. It is worth stating that understanding the behavior of nanoparticles under peristaltic flow conditions could aid in the development of more efficient drug delivery systems, the design and optimization of nanofluidic devices, improved mixing performance in microfluidic devices, and the design of heat transfer systems with improved performance. Overall, this study aims to contribute to the comprehensive understanding of the peristaltic flow of hybrid nanofluids and its applications in biomedical technology and biomedicine.

## 2. Problem Formulation

**Figure 1:**
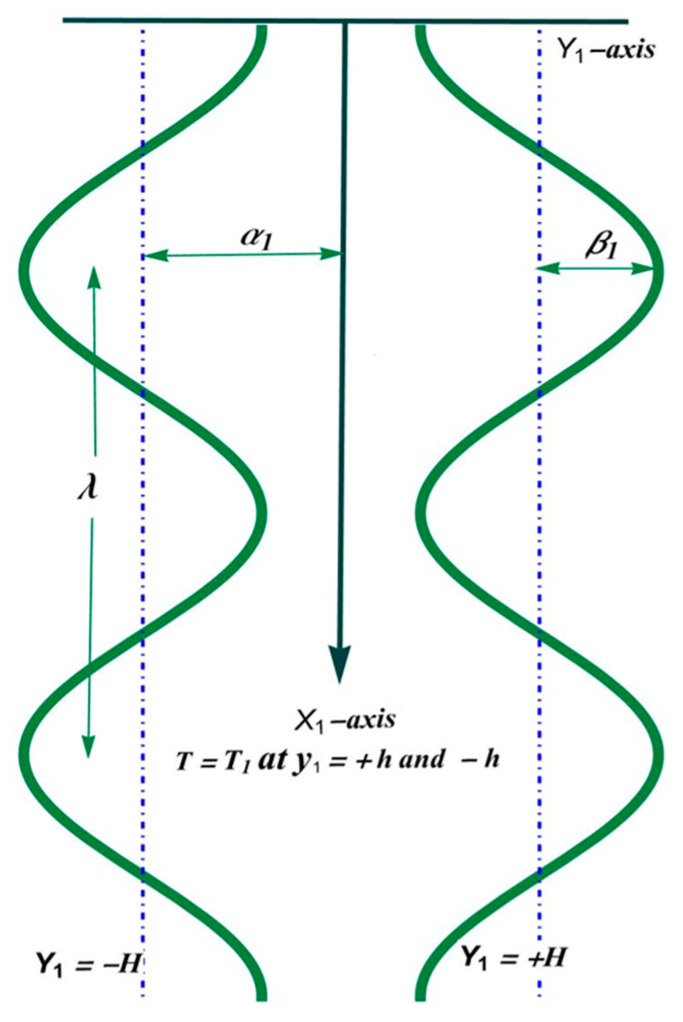
The flow configuration.

A 2D incompressible peristaltic transport of an electrically conducting pattern of hybrid nanoparticles (MWCNT−ZnO/engine oil), due to a symmetric channel having width ‘$2\alpha_{1}$’, is considered. The sinusoidal waves propagate with a wavelength of ‘λ’ along its walls at constant wave speed $c_{1}$. Here, the Cartesian coordinates ($\bar{X_{1}}$, $\bar{Y_{1}}$) are employed, $\bar{Y_{1}}-\mathrm{axis}$ and $\bar{X_{1}}-\mathrm{axis}$ are reserved along the perpendicular and parallel to the middle line, respectively (see Figure 1).

The study describes the geometric features of the wall surface as follows:

(1) $$\pm \bar{H}(\bar{X_{1}},\bar{t_{1}})=\pm \beta_{1}\cos\left(\frac{2\pi }{\lambda }(\bar{X_{1}}-c_{1}\bar{t_{1}})\right)\pm \alpha_{2},$$

where $c_{1}$ is the dimensionless speed, $\beta_{1}$ is the amplitude of the peristaltic waves, $\bar{t_{1}}$ is the time, and λ is the wavelength, respectively. Furthermore, $-\bar{H}(\bar{X_{1}},\bar{t_{1}})$ and $+\bar{H}(\bar{X_{1}},\bar{t_{1}})$ are symbolized to show the geometry of the stated problem for lower and upper walls. The Lorentz force is described as:

(2) $$\vec{F_{2}}=\vec{J_{2}}\times \vec{B_{2}}.$$

The velocity field for current flow behavior is denoted as $\vec{V}=\left[\bar{U_{1}}\left(\bar{X_{1}},\bar{Y_{1}},\bar{t_{1}}\right),\bar{V_{1}}\left(\bar{X_{1}},\bar{Y_{1}},\bar{t_{1}}\right),0\right]$, where $\vec{J_{2}}$ represents the current density and $\bar{B_{2}}=(0,0,B_{1})$ is the applied magnetic field. The “Ohm’s law with Hall effects” is defined as [2]:

(3) $$\vec{J_{2}}=\sigma_{hnf}\left\{\vec{V}\times \vec{B_{2}}+\vec{E_{2}}-\frac{\left(\vec{J_{2}}\times \vec{B_{2}}\right)}{e_{1}.n_{e_{1}}}\right\}.$$

In the above equation, $\vec{V}$, $\vec{B_{2}}$, $\sigma_{hnf}$, $\vec{E_{2}}$, and $n_{e_{1}}$ imply the velocity field, magnetic field, electric conductivity of hybrid nanofluids, electric field, and free electron density, respectively. If there is no electric field, substituting Equation (3) into Equation (2) results in the following expression:

(4) $$\vec{F_{2}}=\left[\frac{(B_{1}{}^{2})\sigma_{hnf}}{\left(1+{\left(B_{1}\sigma_{hnf}\right)}^{2}/{\left(n_{e_{1}}.e_{1}\right)}^{2}\right)}\left(\left(\frac{B_{1}\sigma_{hnf}}{n_{e_{1}}.e_{1}}\right)\bar{V_{1}}-\bar{U_{1}}\right),-\frac{B_{1}{}^{2}\sigma_{hnf}}{\left(1+{\left(B_{1}\sigma_{hnf}\right)}^{2}/{\left(n_{e_{1}}.e_{1}\right)}^{2}\right)}\left(\left(\frac{B_{1}\sigma_{hnf}}{n_{e_{1}}.e_{1}}\right)\bar{U_{1}}+\bar{V_{1}}\right),0\right].$$

The study presents the expression for the electric conductivity of hybrid nanofluids [2]. This equation mathematically describes the relationship between the electric conductivity of the fluid and various parameters, including the concentration of nanoparticles, the electric conductivity of the nanoparticles, and the electric conductivity of the base fluid.

(5) $$\frac{\sigma_{hnf}}{\sigma_{f}}=1+\frac{3\left(\frac{\sigma_{M}\phi_{M}+\sigma_{Z}\phi_{Z}}{\sigma_{f}}\right)-3\left(\phi_{M}+\phi_{Z}\right)}{\left(\frac{\sigma_{M}\phi_{M}+\sigma_{Z}\phi_{Z}}{\sigma_{f}\left(\phi_{M}+\phi_{Z}\right)}+2\right)-\left(\frac{\sigma_{M}\phi_{M}+\sigma_{Z}\phi_{Z}}{\sigma_{f}}\right)-\left(\phi_{M}+\phi_{Z}\right)}=B_{4}$$

Here, ‘$\phi_{M}$’ is the volume frictions of *MWCNT*, ‘$\phi_{Z}$’ is the nano-particles volume friction of zinc oxide, and ‘$\sigma_{f}$’ is the electric conductivity for base fluid. The expression for the Lorentz force is obtained by substituting Equation (5) into Equation (4), as shown in the study. This mathematical operation enables the relationship between the Lorentz force and other parameters to be analyzed and understood.

(6) $$\vec{F_{2}}=\left[\frac{B_{4}B_{2}{}^{2}}{B_{4}{}^{2}m_{1}^{2}+1}\left(-\bar{U_{1}}+m_{1}B_{4}\bar{V_{1}}\right)\sigma_{f},-\frac{\sigma_{f}B_{4}B_{2}{}^{2}}{B_{4}{}^{2}m_{1}^{2}+1}\left(m_{1}B_{4}\bar{U_{1}}+\bar{V_{1}}\right),0\right],m_{1}=\frac{B_{2}\sigma_{f}}{n_{e}.e}$$

where $m_{1}$ is the Hall parameter, while the expression for Ohmic heating is indicated as under [2]:

(7) $$\frac{1}{\sigma_{hnf}}\vec{J_{2}}\cdot \vec{J_{2}}=\frac{\sigma_{f}B_{4}B_{1}{}^{2}}{1+B_{4}{}^{2}m_{1}^{2}}\left(\bar{U_{1}^{2}}+\bar{V_{1}^{2}}\right).$$

References [2,3,4,5,6,7,8,9] contain the descriptions of the equations that govern the current flow, which are mentioned below:

(8) $$\frac{\partial \bar{V_{1}}}{\partial \bar{Y_{1}}}+\frac{\partial \bar{U_{1}}}{\partial \bar{X_{1}}}=0,$$

(9) $$\left[\frac{\partial \bar{U_{1}}}{\partial \bar{t_{1}}}+\bar{U_{1}}\frac{\partial \bar{U_{1}}}{\partial \bar{X_{1}}}+\bar{V_{1}}\frac{\partial \bar{U_{1}}}{\partial \bar{Y_{1}}}\right]=-\frac{\partial \bar{P_{1}}}{\partial \bar{X_{1}}}+\mu_{hnf}\left[\frac{\partial^{2}\bar{U_{1}}}{\partial^{2}\bar{Y_{1}}}+\frac{\partial^{2}\bar{U_{1}}}{\partial^{2}\bar{X_{1}}}\right]\;-\frac{\sigma_{f}B_{4}{B_{1}}^{2}}{1+{B_{4}}^{2}m_{1}^{2}}\left(\bar{U_{1}}-m_{1}B_{4}\bar{V_{1}}\right)+g{\left(\rho \beta \right)}_{hnf}\left(T-T_{1}\right),$$

(10) $$\rho_{hnf}[\left[\frac{\partial \bar{V_{1}}}{\partial \bar{t_{1}}}+\bar{U_{1}}\frac{\partial \bar{V_{1}}}{\partial \bar{X_{1}}}+\bar{V_{1}}\frac{\partial \bar{V_{1}}}{\partial \bar{Y_{1}}}\right]=-\frac{\partial \bar{P_{1}}}{\partial \bar{Y1}}\;+\mu_{hnf}\left[\frac{\partial^{2}\bar{V_{1}}}{\partial^{2}\bar{Y_{1}}}+\frac{\partial^{2}\bar{V_{1}}}{\partial \bar{X_{2}}}\right]-\frac{\sigma_{f}B_{4}{B_{1}}^{2}}{1+{B_{4}}^{2}m_{1}^{2}}\left(m_{1}B_{4}\bar{U_{1}}+\bar{V_{1}}\right),$$

(11) $${\left(\rho C\right)}_{hnf}\left[\frac{\partial T}{\partial \bar{t_{1}}}+\bar{U_{1}}\frac{\partial T}{\partial \bar{X_{1}}}+\bar{V_{1}}\frac{\partial T}{\partial \bar{Y_{1}}}\right]=\left(\frac{\partial }{\partial \bar{X_{1}}}+\frac{\partial }{\partial \bar{Y_{1}}}\right)\left(K_{hnf}\left(T\right)\left(\frac{\partial T}{\partial \bar{X_{1}}}+\frac{\partial T}{\partial \bar{Y_{1}}}\right)\right)-\frac{\partial Q_{r}}{\partial \bar{Y_{1}}}+\Phi_{1}\;+\frac{B_{4}\sigma_{f}{B_{1}}^{2}}{1+{B_{4}}^{2}m_{1}^{2}}\left(\bar{V_{1}^{2}}+\bar{U_{1}^{2}}\right)+\mu_{hnf}\left[{\left(\frac{\partial \bar{V_{1}}}{\partial \bar{X_{1}}}+\frac{\partial \bar{U_{1}}}{\partial \bar{Y1}}\right)}^{2}+\left(2{\left(\frac{\partial \bar{U1}}{\partial \bar{X1}}\right)}^{2}+2{\left(\frac{\partial \bar{V1}}{\partial \bar{Y1}}\right)}^{2}\right)\right].$$

In the above equations, $\Phi_{1}$ is the heat absorption/generation parameter, $\bar{P_{1}}\left(\bar{X_{1}},\bar{Y_{1}},{\bar{t}}_{1}\right)$ is the pressure, $\beta_{hnf}$ is the thermal expansion coefficient, $\rho_{f}$ is the density of nanofluid, $\mu_{hnf}$, $C_{hnf}$, $\rho_{hnf}$, and $K_{hnf}$ are the viscosity, specific heat, density, and thermal conductivity, respectively, of the hybrid nanofluid. The temperature-dependent thermal conductivity [10] with parameter α is defined as:

(12) $$K_{f}=K_{1}\left(1+\alpha (T-T_{1})\right).$$

The radiative heat flux is approximated using Rosseland’s approximation in the study [11], i.e.,

(13) $$Q_{r}=-\frac{16}{3}\frac{\sigma_{1}^{*}}{k_{1}^{*}}T_{1}{}^{3}\frac{\partial T}{\partial \bar{Y_{1}}}.$$

Here, $k_{1}^{*}$ is the Rosseland mean absorption and $\sigma_{1}^{*}$ represents the Stefan-Boltzmann constant. The thermo-physical properties of the hybrid nanofluids considered in this study are based on the values provided in reference [13]. Thus, we have

(14) $$\left\{ \begin{array}{l} \rho_{hnf}=\rho_{M}\phi_{M}+\left(1-\left(\phi_{M}+\phi_{Z}\right)\right)\rho_{f}+\rho_{Z}\phi_{Z},\;\\ {\left(\rho C\right)}_{hnf}={\left(\rho C\right)}_{Z}\phi_{Z}+\left(1-\left(\phi_{Z}+\phi_{M}\right)\right){\left(\rho C\right)}_{f}+{\left(\rho C\right)}_{M}\phi_{M},\\ {\left(\rho \beta \right)}_{hnf}={\left(\rho \beta \right)}_{Z}\phi_{Z}+\left(1-\left(\phi_{M}+\phi_{Z}\right)\right){\left(\rho \beta \right)}_{f}+\phi_{M}{\left(\rho \beta \right)}_{M},\\ \frac{K_{hnf}}{K_{nf}}=\frac{K_{z}+2K_{nf}-2\phi_{Z}\left(K_{nf}-K_{Z}\right)}{K_{z}+2K_{nf}+2\phi_{Z}\left(K_{nf}-K_{Z}\right)},\\ \frac{\mu_{hnf}}{\mu_{f}}={\left(1-\phi_{M}\right)}^{-2.5}{\left(1-\phi_{Z}\right)}^{-2.5},\\ \frac{K_{nf}}{K_{f}}=\frac{K_{M}+2K_{f}-2\phi_{M}\left(K_{f}-K_{M}\right)}{K_{M}+2K_{f}+\phi_{M}\left(K_{f}-K_{M}\right)}. \end{array}\right.$$

The lower indices f, M, nf, and Z signify the base fluid, *MWCNT*, nanofluid, and zinc oxide (ZnO), correspondingly. The tabular values of thermophysical factors for MWCNT, ZnO, and an engine oil are provided in Table 1, where units of *ρ*, *K*, *C*, *σ*, and *β* are $Kgm^{-3}$, $Wm^{-1}k^{-1}$, $J{\left(Kg\right)}^{-1}K^{-1}$, $Sm^{-1}$, and $k^{-1}$, respectively.

The relationship between fixed frame $\left(\bar{X_{1}},\bar{Y_{1}},\bar{t_{1}}\right)$ and wave frame $\left(\bar{x_{1}},\bar{y_{1}}\right)$ is given as [1]:

(15) $$\begin{array}{c} \bar{v_{1}}\left(\bar{x_{1}},\bar{y_{1}}\right)=\bar{V_{1}}\left(\bar{X_{1}},\bar{Y_{1}},\bar{t_{1}}\right),\;\bar{p_{1}}\left(\bar{x_{1}},\bar{y_{1}}\right)=\bar{P_{1}}\left(\bar{X_{1}},\bar{Y_{1}},\bar{t_{1}}\right),\;\bar{x_{1}}=\bar{X_{1}}-c_{1}\bar{t_{1},}\\ \bar{u_{1}}\left(\bar{X_{1}},\bar{y_{1}}\right)=\bar{U_{1}}\left(\bar{X_{1}},\bar{Y_{1}},\bar{t_{1}}\right)-c_{1},\;\bar{Y_{1}}=\bar{y_{1}}. \end{array}$$

Utilizing the above-stated transformation to Equations (8)–(11), it is found that

(16) $$\frac{\partial \bar{u_{1}}}{\partial \bar{x_{1}}}+\frac{\partial \bar{v_{1}}}{\partial \bar{y_{1}}}=0,$$

(17) $$\begin{array}{l} \left(\left(1-\left(\phi_{Z}+\phi_{W}\right)\right)\rho_{f}+\phi_{Z}\rho_{Z}+\phi_{W}\rho_{W}\right)\left[\bar{v_{1}}\;\frac{\partial \left[\left(\bar{u_{1}}+c_{1}\right)\right]}{\partial \bar{y_{1}}}+\frac{\partial \left[\left(\bar{u_{1}}+c_{1}\right)\right]}{\partial \bar{x_{1}}}\left(\bar{u_{1}}+c_{1}\right)\right]=-\frac{\partial \bar{p_{1}}}{\partial \bar{x_{1}}}\\ +\mu_{f}{\left(1-\phi_{Z}\right)}^{-2.5}{\left(1-\phi_{W}\right)}^{-2.5}\left[\frac{\partial^{2}\bar{u_{1}}}{\partial {\bar{y}}_{1}^{2}}+\frac{\partial^{2}\bar{u_{1}}}{\partial {\bar{x}}_{1}^{2}}\right]-\frac{\sigma_{f}B_{4}B_{2}{}^{2}}{1+B_{4}{}^{2}m_{1}^{2}}\left[\left(\bar{u_{1}}+c_{1}\right)-mB_{4}\bar{v_{1}}\right]+g{\left(\rho \beta \right)}_{hnf}\left(T-T_{1}\right), \end{array}$$

(18) $$\begin{array}{l} \left(\left(1+\left(\phi_{Z}-\phi_{W}\right)\right){\left(\rho C\right)}_{f}+\phi_{W}{\left(\rho C\right)}_{W}+\phi_{Z}{\left(\rho C\right)}_{Z}\right)\left[\bar{v_{1}}\frac{\partial T}{\partial \bar{y_{1}}}+\left(\bar{u_{1}}+c_{1}\right)\frac{\partial T}{\partial \bar{x_{1}}}\right]=\frac{16}{3}\frac{\sigma^{*}}{k^{*}}T_{0}{}^{3}\frac{\partial^{2}T}{\partial \bar{y_{2}}}+\frac{B_{4}\sigma_{f}B_{2}^{2}}{1+B_{4}{}^{2}m^{2}}\left({\left(\bar{u_{1}}+c_{1}\right)}^{2}+\bar{v_{1}^{2}}\right)\\ +\left[\frac{\partial T}{\partial \bar{y_{1}}}+\frac{\partial T}{\partial \bar{x_{1}}}\right]\left(K_{f}\left[\frac{K_{z}+2\left(\frac{K_{W}+2K_{f}-2\phi_{W}\left(K_{f}-K_{W}\right)}{K_{W}+2K_{f}+\phi_{W}\left(K_{f}-K_{W}\right)}\right)-2\phi_{Z}\left(\frac{K_{W}+2K_{f}-2\phi_{W}\left(K_{f}-K_{W}\right)}{K_{W}+2K_{f}+\phi_{W}\left(K_{f}-K_{W}\right)}-K_{Z}\right)}{K_{z}+2\left(\frac{K_{W}+2K_{f}-2\phi_{W}\left(K_{f}-K_{W}\right)}{K_{W}+2K_{f}+\phi_{W}\left(K_{f}-K_{W}\right)}\right)+2\phi_{Z}\left(\frac{K_{W}+2K_{f}-2\phi_{W}\left(K_{f}-K_{W}\right)}{K_{W}+2K_{f}+\phi_{W}\left(K_{f}-K_{W}\right)}-K_{Z}\right)}\right]\left[\frac{\partial T}{\partial \bar{y_{1}}}+\frac{\partial T}{\partial \bar{x_{1}}}\right]\right)\\ +\Phi_{1}+\frac{\mu_{f}}{{\left(1-\phi_{W}\right)}^{2.5}{\left(1-\phi_{Z}\right)}^{2.5}}\left[2\left({\left(\frac{\partial \bar{u_{1}}}{\partial \bar{x_{1}}}\right)}^{2}+{\left(\frac{\partial \bar{v_{1}}}{\partial \bar{y_{1}}}\right)}^{2}\right)+{\left(\frac{\partial \bar{u_{1}}}{\partial \bar{y_{1}}}+\frac{\partial \bar{v_{1}}}{\partial \bar{x_{1}}}\right)}^{2}\right]. \end{array}$$

Below is a list of the flow variables or parameters that are dimensionless:

(19) u1=u1¯c1,x1=x1¯λ,h=H¯α1,a=β1α1,p1=α12p1¯c1λμf, u1=∂ψ∂y1,θ=T−T1T1,Gr=ρfβfgT1α12μfc1,M1=σfμfB1α1,Re=ρfc1α1μf,Pr=μfCfKf, y1=y1¯α1, v1=v1¯c1δ,Nr=163σ1∗k1∗T13Kf,E=c12CfT1, Br=PrE,v1=−∂ψ∂x1,ε=α22Φ1T1Kf.

With the small Reynolds number and long wavelength approximations, Equations (16)–(19) can be expressed in the following form:

(20) $$0=-\frac{\partial p_{1}}{\partial x_{1}}+B_{2}Gr\theta +B_{1}\frac{\partial^{3}\psi }{\partial y_{1}^{3}}-\left\{\frac{B_{4}M_{1}^{2}}{B_{4}{}^{2}m_{1}^{2}+1}\right\}\left[1+\frac{\partial \psi }{\partial y_{1}}\right],$$

(21) $$0=\frac{\partial p_{1}}{\partial y_{1}},$$

(22) $$\begin{array}{c} 0=\frac{Br}{{\left(1-\phi_{W}\right)}^{2.5}{\left(1-\phi_{Z}\right)}^{2.5}}{\left(\frac{\partial^{2}\psi }{\partial y_{1}^{2}}\right)}^{2}+B_{3}\frac{\partial }{\partial y_{1}}\left(\left(1+\alpha \theta \right)\frac{\partial \theta }{\partial y_{1}}\right)\\ +\left\{\frac{B_{4}\left(BrM_{1}^{2}\right)}{1+m_{1}^{2}B_{4}^{2}}\right\}\left[{\left(1+\frac{\partial \psi }{\partial y_{1}}\right)}^{2}\right]+Nr\frac{\partial^{2}\theta }{\partial y_{1}^{2}}+\varepsilon . \end{array}$$

Applying cross differentiating, Equations (21) and (22) yield:

(23) $$0=B_{1}\frac{\partial^{4}\psi }{\partial y_{1}^{4}}+\left(B_{2}Gr\right)\frac{\partial \theta }{\partial y_{1}}-\left(\frac{B_{4}M^{2}}{1+B_{4}{}^{2}m^{2}}\right)\left(\frac{\partial^{2}\psi }{\partial y_{1}^{2}}\right).$$

Here, ε, Gr, Br, ψ, Pr, a, Re, Nr, $M_{1}$, θ, and $m_{1}$ are characterized heat absorption/generation parameter, the Grashof number, the Brinkman number, stream function, the Prandtl number, the temperature-dependent thermal conductivity parameter, the Reynolds number, the thermal radiation parameter, the Hall parameter, the dimensionless temperature, and the Hartman number, respectively. The two-dimensional stream function, as defined in Equation (20), is considered in the form as its introduction identically satisfies Equation (16), see for a reference [22]. The constants $B_{1},B_{2}$, and $B_{3}$ are defined as:

(24) $$\left\{ \begin{array}{l} \frac{{\left(\rho \beta \right)}_{hnf}}{{\left(\rho \beta \right)}_{f}}=1-\left(\phi_{Z}+\phi_{M}\right)+{\frac{1}{\left(\rho \beta \right)}}_{f}\left\{\phi_{Z}{\left(\rho \beta \right)}_{Z}+\phi_{M}{\left(\rho \beta \right)}_{M}\right\}=B_{2},\\ \frac{K_{hnf}}{K_{f}}=\left[\frac{K_{z}+2\left(\frac{K_{M}+2K_{f}-2\phi_{M}\left(K_{f}-K_{M}\right)}{K_{M}+2K_{f}+\phi_{M}\left(K_{f}-K_{M}\right)}\right)-2\phi_{Z}\left(\frac{K_{M}+2K_{f}-2\phi_{M}\left(K_{f}-K_{M}\right)}{K_{M}+2K_{f}+\phi_{M}\left(K_{f}-K_{M}\right)}-K_{Z}\right)}{K_{z}+2\left(\frac{K_{M}+2K_{f}-2\phi_{M}\left(K_{f}-K_{M}\right)}{K_{M}+2K_{f}+\phi_{M}\left(K_{f}-K_{M}\right)}\right)+2\phi_{Z}\left(\frac{K_{M}+2K_{f}-2\phi_{M}\left(K_{f}-K_{M}\right)}{K_{M}+2K_{f}+\phi_{M}\left(K_{f}-K_{M}\right)}-K_{Z}\right)}\right]=B_{3}\\ B_{1}={\left(1-\phi_{Z}\right)}^{-2.5}{\left(1-\phi_{M}\right)}^{-2.5}. \end{array}\right.$$

The boundary conditions are expressed in a dimensionless form, as shown below:

(25) $$\begin{array}{c} \psi =0,\frac{\partial \theta }{\partial y_{1}}=0,\frac{\partial^{2}\psi }{\partial y_{1}^{2}}=0,{\;\mathrm{at}\;y}_{1}=0,\\ \frac{\partial \psi }{\partial y_{1}}+\frac{\beta_{2}}{{\left(1-\phi_{M}\right)}^{2.5}{\left(1-\phi_{Z}\right)}^{2.5}}\frac{\partial^{2}\psi }{\partial y_{1}^{2}}=-1,\psi =F,\\ \left[\frac{K_{z}+2\left(\frac{K_{M}+2K_{f}-2\phi_{M}\left(K_{f}-K_{M}\right)}{K_{M}+2K_{f}+\phi_{M}\left(K_{f}-K_{M}\right)}\right)-2\phi_{Z}\left(\frac{K_{M}+2K_{f}-2\phi_{M}\left(K_{f}-K_{M}\right)}{K_{M}+2K_{f}+\phi_{M}\left(K_{f}-K_{M}\right)}-K_{Z}\right)}{K_{z}+2\left(\frac{K_{M}+2K_{f}-2\phi_{M}\left(K_{f}-K_{M}\right)}{K_{M}+2K_{f}+\phi_{M}\left(K_{f}-K_{M}\right)}\right)+2\phi_{Z}\left(\frac{K_{M}+2K_{f}-2\phi_{M}\left(K_{f}-K_{M}\right)}{K_{M}+2K_{f}+\phi_{M}\left(K_{f}-K_{M}\right)}-K_{Z}\right)}\right]\left(\frac{\partial \theta }{\partial y_{1}}\right)+Bi\theta =0,\;\mathrm{at}\;y_{1}=h, \end{array}$$

The peristaltic wall boundary conditions are specified using a dimensionless form, which is given by:

(26) $$\pm h(x_{1})=\pm 1\pm a\cos(2\pi x_{1}).$$

Here, the parameter “Bi (=$\frac{l\alpha_{2}}{K_{f}}$)” represents the Biot number, l being the heat transfer coefficient, whereas the parameter “$\beta_{2}$ (=$\frac{\mu_{f}\beta_{3}}{\alpha_{2}}$)” represents the dimensionless velocity slip parameter and $\beta_{3}$ the dimensional slip parameter.

## 3. Expressions for Entropy Generation

The expressions for entropy generation in dimensional forms, as given in reference [2], can be expressed as follows:

(27) $$S_{G}=\frac{K_{hnf}\left(T\right)}{T_{1}{}^{2}}\left\{{\left(\bar{\nabla }T\right)}^{2}\right\}+\left\{\frac{\mu_{hnf}}{T_{1}}\varphi \right\}+\left(\left(\bar{U_{1}^{2}}+\bar{V_{1}^{2}}\right)\;\frac{B_{4}\sigma_{f}B_{1}^{2}}{1+B_{4}^{2}m_{1}^{2}}\right)+\frac{1}{T_{1}^{2}}\frac{16}{3}\frac{\sigma_{1}^{*}}{k_{1}^{*}}T_{1}^{3}{\left(\frac{\partial T}{\partial \bar{Y_{1}}}\right)}^{2},$$

where

(28) $$\varphi =\left[{\left(\frac{\partial \bar{V_{1}}}{\partial \bar{X_{1}}}+\frac{\partial \bar{U_{1}}}{\partial \bar{Y_{1}}}\right)}^{2}\right]+\left\{2{\left(\frac{\partial \bar{V_{1}}}{\partial \bar{Y_{1}}}\right)}^{2}+2{\left(\frac{\partial \bar{U_{1}}}{\partial \bar{X_{1}}}\right)}^{2}\right\}.$$

The entropy generation number $N_{S}$ is the fraction of the entropy generation rate in actuality and the specific entropy generation rate, as defined by the subsequent equation:

$$N_{S}=\frac{S_{G}}{S_{c}}.$$

The “non-dimensional formula of entropy generation number” is stated as:

(29) $$N_{S}=B_{1}Br{\left(\frac{\partial^{2}\psi }{\partial y^{2}}\right)}^{2}+\left[Br\left(\frac{B_{4}M_{1}^{2}}{1+B_{4}m_{1}^{2}}\right)\right]\left[{\left(\frac{\partial \psi }{\partial y_{1}}+1\right)}^{2}\right]+B_{3}\left(1+\alpha \theta \right){\left(\frac{\partial \theta }{\partial y_{1}}\right)}^{2}+Nr{\left(\frac{\partial \theta }{\partial y_{1}}\right)}^{2}.$$

A graphical description of the results is discussed in the next section.

## 4. Solution Methodology

To obtain solutions for Equations (23), (24), and (30) in their dimensionless forms, the HAM is used. To use this method, suitable initial approximations and a linear operator must be obtained.

(30) $$\psi_{0}(y_{1})=\frac{h^{3}y_{1}-hy_{1}^{3}+f_{0}\left(6B_{1}hy_{1}+3h^{2}y_{1}-y_{1}^{3}\right)}{2h^{2}\left(3B_{1}+h\right)},$$

(31) $$\theta_{0}(y_{1})=\frac{\left(Bih^{2}-Bi\;y_{1}^{2}+2hB_{3}\right)}{2Bi(B_{3}+Nr)},$$

(32) $$L_{\psi }=B_{1}\frac{\partial^{4}}{\partial y_{1}^{4}},$$

(33) $$\begin{array}{l} \;\psi_{0}=0,\psi_{0}^{\prime \prime }=0,{\;\mathrm{at}\;y}_{1}=0,\\ \psi_{0}^{’}+A_{1}\psi_{0}^{\prime \prime }=-1,\psi =f_{0}, \end{array}$$

(34) $$L_{\theta }=B_{3}\frac{\partial^{2}}{\partial y_{1}^{2}}+Nr\frac{\partial^{2}}{\partial y_{1}^{2}}+\varepsilon ,$$

(35) $$\;\theta_{0}=0,\;B_{3}\theta_{0}^{\prime \prime }+Bi\theta_{0}=0,{\;\mathrm{at}\;y}_{1}=0.$$

### 4.1. Zeroth-Order Deformation

To determine the behavior of a system under small perturbations. The zeroth order deformation is given by

(36) $$(1-p)L_{\psi }\left[\psi (y_{1};p)-\psi_{0}(y_{1},p)\right]=pq_{\psi }H_{\psi }[\theta (y_{1};p),\psi (y_{1};p)],\;(1-p)L_{\theta }\left[\theta (y_{1};p)-\theta_{0}(y_{1},p)\right]=pq_{\theta }H_{\theta }[\psi (y_{1};p),\theta (y_{1};p)],$$

here, p∈[0,1] is the embedding parameter, $H_{\psi }$ and $H_{\theta }$ are the auxiliary parameters, $q_{\theta }$ and $q_{\psi }$ symbolize nonlinear operators, L is an auxiliary linear operator. The initial approximation $\theta_{0}(y_{1},p)$ and $\psi_{0}(y_{1},p)$ approach $\theta (y_{1},p)$ and $\psi (y_{1},p)$, respectively, as the p value taken from 0 to 1. Mathematically,

(37) $$\begin{array}{ll} \psi (y_{1},0)=\psi_{0}(y_{1}), & \psi (y_{1},1)=\psi (y_{1}),\\ \theta (y_{1},0)=\theta_{0}(y_{1}), & \theta (y_{1},1)=\theta (y_{1}). \end{array}$$

### 4.2. Nth-Order Deformation

The Nth order deformation is given by

(38) $$\begin{array}{l} L_{\theta }\left[\theta_{N}\left(y_{1}\right)-\zeta_{N}\theta_{N-1}\left(y_{1}\right)\right]=H_{\theta }R_{N}^{\theta }\left(y_{1}\right),\\ L_{\psi }\left[\psi_{N}\left(y_{1}\right)-\zeta_{N}\psi_{N-1}\left(y_{1}\right)\right]=H_{\psi }R_{N}^{\psi }\left(y_{1}\right). \end{array}$$

with

(39) $$\begin{array}{l} R_{N}^{\theta }(y_{1})={\frac{1}{\left(N-1\right)!}\frac{\partial^{N-1}q_{\theta }\left[\theta (y_{1},p),\psi (y_{1},p)\right]}{\partial p^{N-1}}|}_{p=0},\\ R_{N}^{\psi }(y_{1})={\frac{1}{\left(N-1\right)!}\frac{\partial^{N-1}q_{\psi }\left[\psi (y_{1},p)\right]}{\partial p^{N-1}}|}_{p=0}.\; \end{array}$$

where

(40) $$\zeta_{N}=\left\{ \begin{array}{l} 0,N\leq 1\\ 1,N>1. \end{array}\right.$$

Thus, the HAM has been exercised to achieve analytical solutions in the form of series expansions. One common approach is to use numerical methods to solve the resulting nonlinear algebraic equations that arise from the HAM solution process. For example, the Newton-Raphson method or the bisection method can be used to solve these equations. However, symbolic computation using Mathematica software has been used here to perform the necessary calculations.

## 5. HAM Solution Convergence

**Figure 2:**
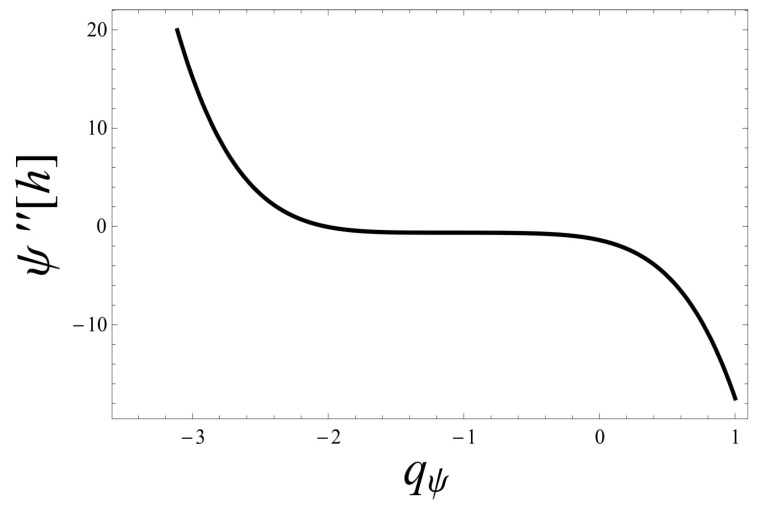
Approximation curve for $\psi^{\prime \prime }[h]$ using the 5th-Order HAM.

**Figure 3:**
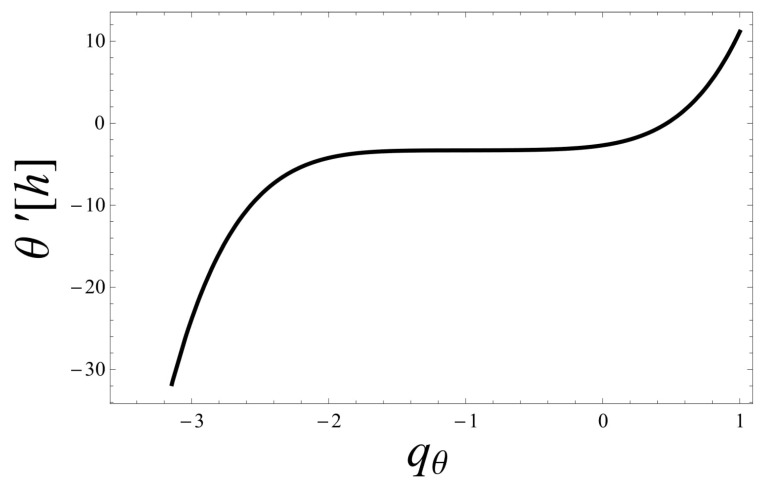
Approximation curve for $\theta^{\prime }[h]$ using the 5th-Order HAM.

Using the HAM, a series solution is constructed by taking suitable values for the auxiliary factors $q_{\psi }$ and $q_{\theta }$. The convergence of the solution heavily relies on the selection of these parameters, which play a significant role in achieving convergence of the series solution. Plotting the q-curve at 5th order-approximation to discover the appropriate values of $q_{\psi }$ and $q_{\theta }$ (see Figure 2 and Figure 3). By examining Figure 2 and Figure 3, it is evident that the appropriate ranges for the auxiliary parameters $-2.2<q_{\psi }<0.2$ and $-2.1<q_{\theta }<0.1$ are provided, respectively, with the convergence of the series solution for $y_{1}=h_{1}$ and $y_{2}=h_{2}$ when $p=1,\;\beta_{2}=0.1,\;x=0,\;\eta =0.6,$ $M_{1}=1.0,\;m_{1}=1.0,$ Nr=1.0, ε=2.0, α=0.03, Br=0.3, Bi=0.5.

## 6. Results and Discussion

This section examines the peristaltic movement of hybrid nanofluids in a symmetric channel due to the influence of a magnetic field, with the channel containing engine oil as the working fluid. The focus is on analyzing the actions of various flow parameters on key transport phenomena, and similarly, velocity and temperature profiles, entropy generation, and heat transfer rates at the channel walls. The findings are presented in the form of graphs to aid in visualization and comprehension. To make the findings more accessible, the analysis is broken down into multiple subsections, each focusing on a specific flow parameter. Overall, this study aims to enhance the understanding of the peristaltic transport of hybrid nanofluids in symmetric channels with the influence of magnetic fields, and to provide insights into the impact of distinct flow factors on the thermodynamic behavior of such systems.

### 6.1. Velocity Profile

**Figure 4:**
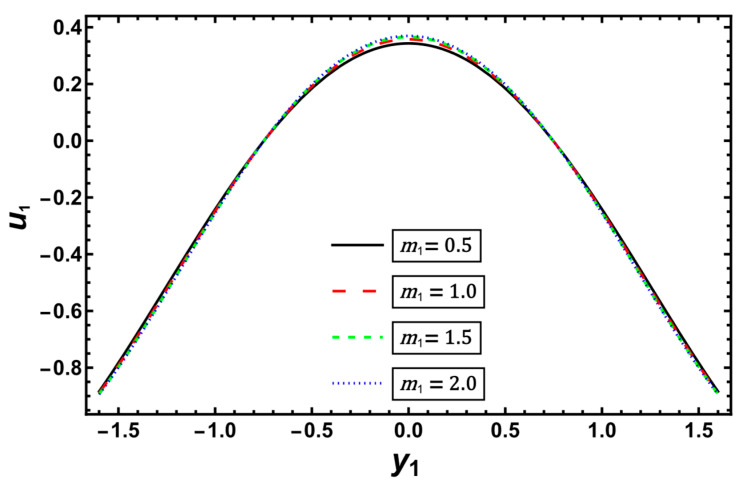
Velocity profile by varying “$m_{1}$” while a=0, x=0, η=0.8, α=0.03, $Bi=0.5,\phi_{M}=0.02,\;\varepsilon =2.0,\;\beta_{2}=0.1,\;M_{1}=1.0,\;\phi_{Z}=0.03,\;Br=0.3,\;Nr=1.0,\;Gr=2.0$.

**Figure 5:**
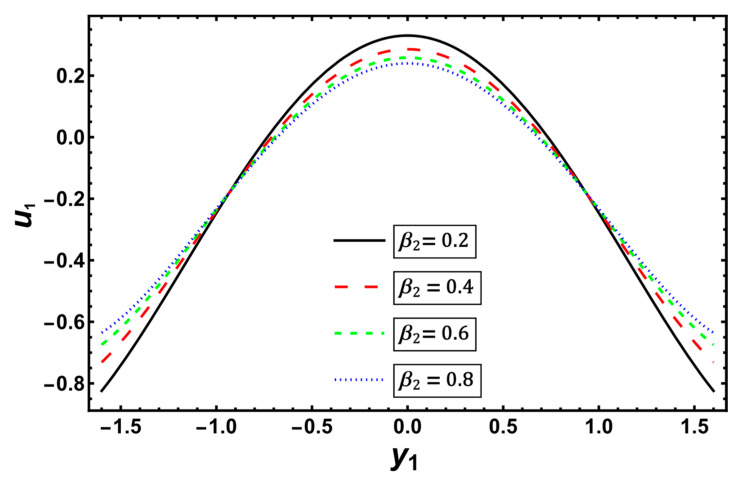
Velocity profile by varying “$\beta_{2}$” while a=0, x=0, η=0.8, α=0.03, $Bi=0.5,\;\phi_{M}=0.02,\;\varepsilon =2.0,\;m_{1}=1.0,\;M_{1}=1.0,\;\phi_{Z}=0.03,\;Br=0.3,\;Nr=1.0,\;Gr=2.0$.

**Figure 6:**
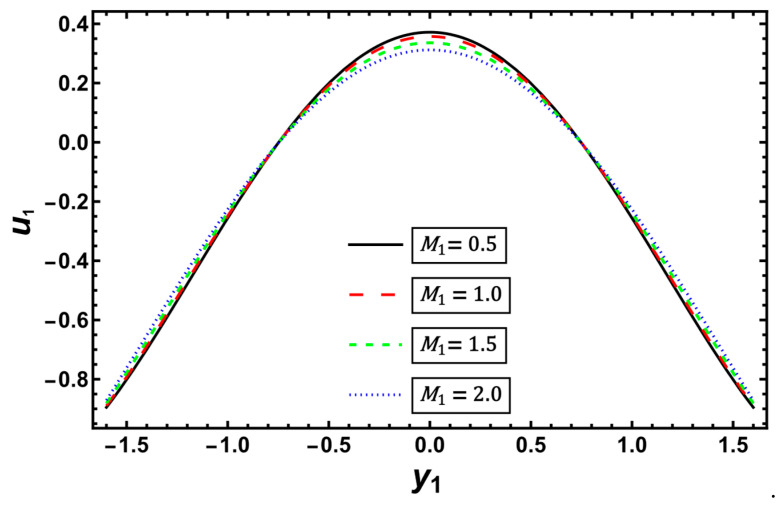
Velocity profile by varying “$M_{1}$” while a=0, x=0, η=0.8, α=0.03, $Bi=0.5,\;\phi_{M}=0.02,\;\varepsilon =2.0,\;\beta_{2}=0.1,\;m_{1}=1.0,\;\phi_{Z}=0.03,\;Br=0.3,\;Nr=1.0,\;Gr=2.0$.

**Figure 7:**
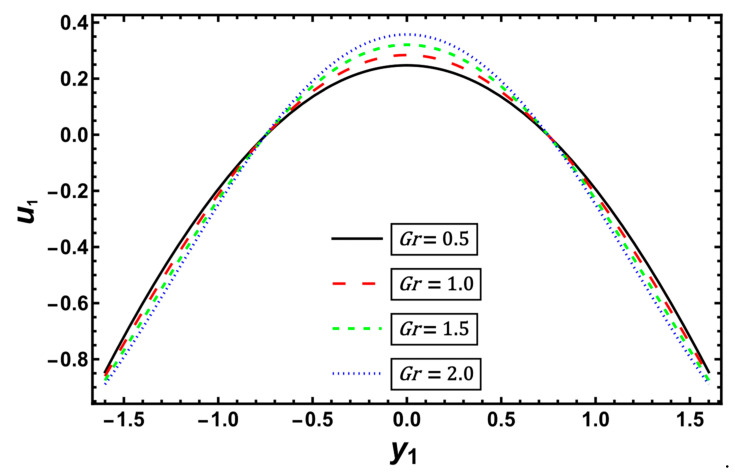
Velocity profile by varying “Gr“ while a=0, x=0, η=0.8, α=0.03, $Bi=0.5,\phi_{M}=0.02,\;\varepsilon =2.0,\;\beta_{2}=0.1,\;M_{1}=1.0,\;\phi_{Z}=0.03,\;Br=0.3,\;Nr=1.0,\;m_{1}=1.0.$

**Figure 8:**
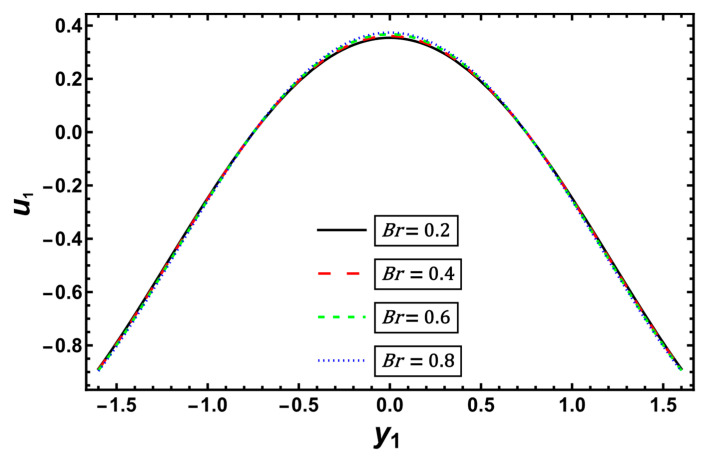
Velocity profile by varying “Br” while a=0, x=0, η=0.8, α=0.03, $Bi=0.5,\phi_{M}=0.02,\;\varepsilon =2.0,\;\beta_{2}=0.1,\;M_{1}=1.0,\;\phi_{Z}=0.03,\;m_{1}=0.3,\;Nr=1.0,\;Gr=2.0.$

**Figure 9:**
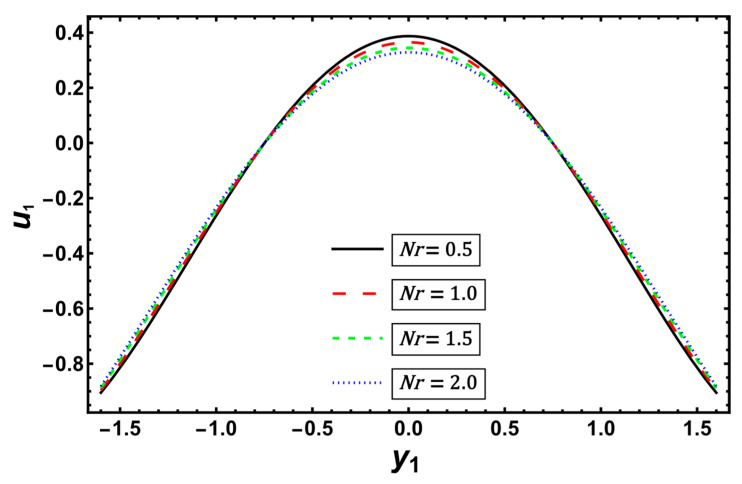
Velocity profile by varying “Nr” while a=0, x=0, η=0.8, α=0.03, $Bi=0.5,\;\phi_{M}=0.02,\;\varepsilon =2.0,\;\beta_{2}=0.1,\;M_{1}=1.0,\;\phi_{Z}=0.03,\;Br=0.3,\;m_{1}=1.0,\;Gr=2.0.$

**Figure 10:**
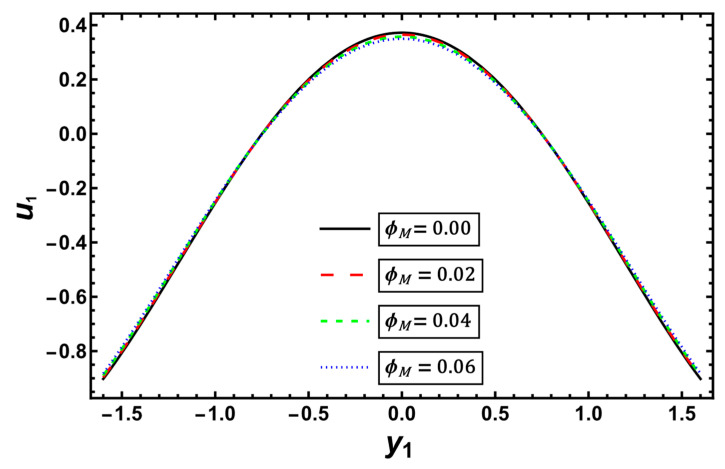
Velocity profile by varying “$\phi_{M}$” while a=0, x=0, η=0.8, α=0.03, $Bi=0.5,\;\varepsilon =2.0,\;\beta_{2}=0.1,\;M_{1}=1.0,\;\phi_{Z}=0.03,\;Br=0.3,\;Nr=1.0,\;Gr=2.0.$

**Figure 11:**
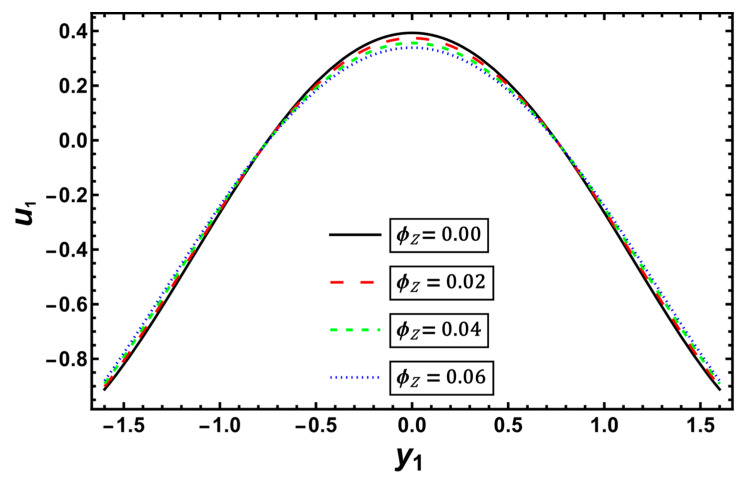
Velocity profile by varying “$\phi_{Z}$” while a=0, x=0, η=0.8, α=0.03, $Bi=0.5,\;\phi_{M}=0.02,\;\varepsilon =2.0,\;\beta_{2}=0.1,\;M_{1}=1.0,\;Br=0.3,\;Nr=1.0,\;Gr=2.0.$

Figure 4, Figure 5, Figure 6, Figure 7, Figure 8, Figure 9, Figure 10 and Figure 11 relate the impact of the Brinkman number (Br), the velocity slip parameter $\left(\beta_{2}\right)$, the Hartman number $\left(M_{1}\right)$, MWCNT nanoparticle volume friction $\left(\phi_{M}\right)$, ZnO nanoparticle volume friction $\left(\phi_{Z}\right)$, the Hall parameter $\left(m_{1}\right)$, the Grashof number (Gr), and the thermal radiation parameter (Nr) on velocity distribution. Figure 4 illustrates that as the value of “$m_{1}$“ rises, there is an improvement in the velocity profile due to the reduction in electrical conductivity production, which results in a decrease in the damping of the magnetic force. Figure 5 depicts the impact of the velocity slip parameter on the velocity distribution, revealing a reduction at the center in the fluid velocity, while the opposite behavior is noticed in both walls for the higher values of $\beta_{2}$. This pattern can be justified by the point that increasing the slip parameter reduces the interaction of the fluid and the walls, which results in a weaker velocity gradient across the channel. As a result, the middle point velocity declines as the slip parameter grows.

Figure 6 displays that as the parameter M increases, there is a drop in the fluid velocity of nanoparticles at the middle point, but an improvement in velocity near the walls. This can be attributed to the acceleration of the Lorentz force caused by the increased magnetic field strength, leading to greater resistance encountered by the fluid particles, and thus, reducing the fluid velocity. Figure 7 displays the relationship between “u” and “Gr”. The velocity adjacent to the central part of the conduit is observed to increase with larger values of the Grashof number, as shown in the figure. Figure 8 represents that axial velocity is boosted in the center of the wall when Br is increased. Figure 9 clearly shows that as the Hartmann number increases, there is a growth in the velocity adjacent to the walls, while near the central point, a decreasing trend is observed. The effects of $‘\phi_{M}’$ and ‘$\phi_{Z}$’ on fluid velocity are demonstrated in Figure 10 and Figure 11, respectively. It is noticed from these figures that both nanoparticles volume friction parameters significantly decrease the hybrid nanofluid velocity distribution in the middle of the walls.

### 6.2. Temperature Profile

**Figure 12:**
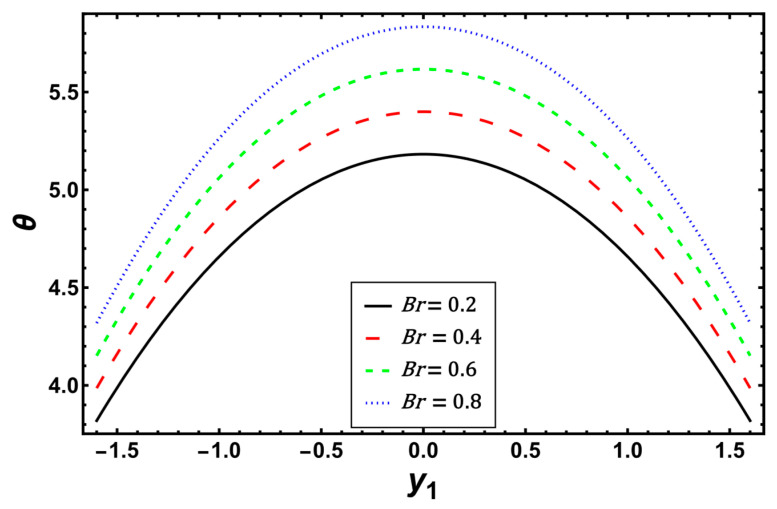
Temperature profile by varying “Br” while a=0, x=0, η=0.8, α=0.03,$Bi=0.5,\;\phi_{M}=0.02,\;\varepsilon =2.0,\;\beta_{2}=0.1,\;M_{1}=1.0,\;\phi_{Z}=0.03,\;m_{1}=1.0,\;Nr=1.0,\;Gr=2.0.$

**Figure 13:**
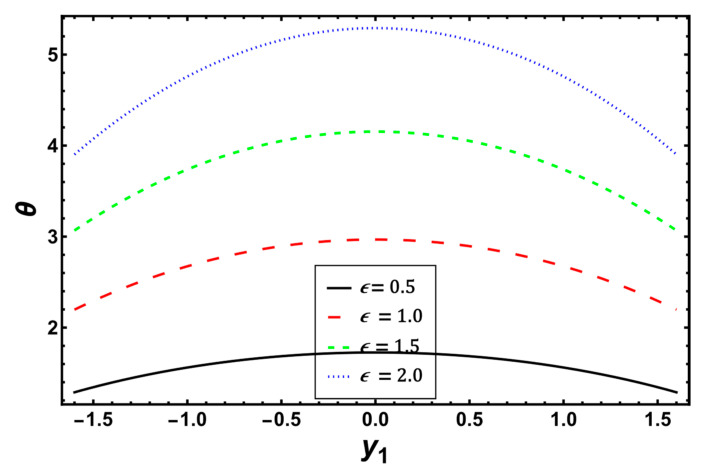
Temperature profile by varying “ε” while a=0, x=0, η=0.8, α=0.03,$Bi=0.5,\;\phi_{M}=0.02,\;\beta_{2}=0.1,\;M_{1}=1.0,\;\phi_{Z}=0.03,\;Br=0.3,\;Nr=1.0,\;Gr=2.0.$

**Figure 14:**
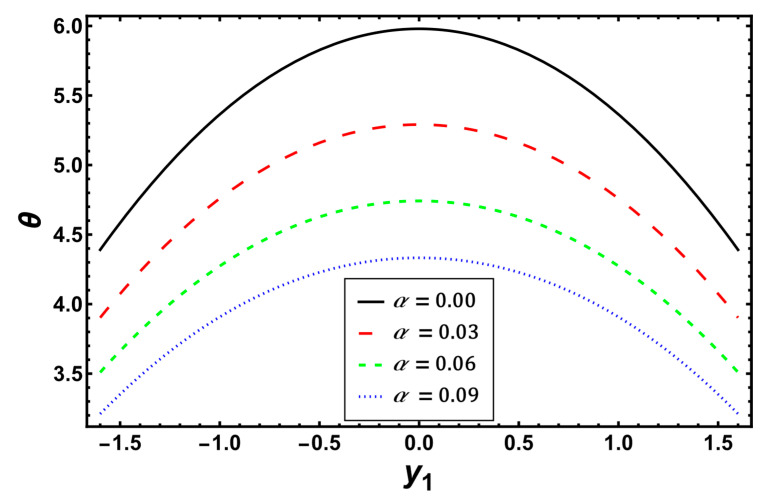
Temperature profile by varying “α” while a=0, x=0, η=0.8,$Bi=0.5,\;\phi_{M}=0.02,\;\varepsilon =2.0,\;\beta_{2}=0.1,\;M_{1}=1.0,\;\phi_{Z}=0.03,\;Br=0.3,\;Nr=1.0.$

**Figure 15:**
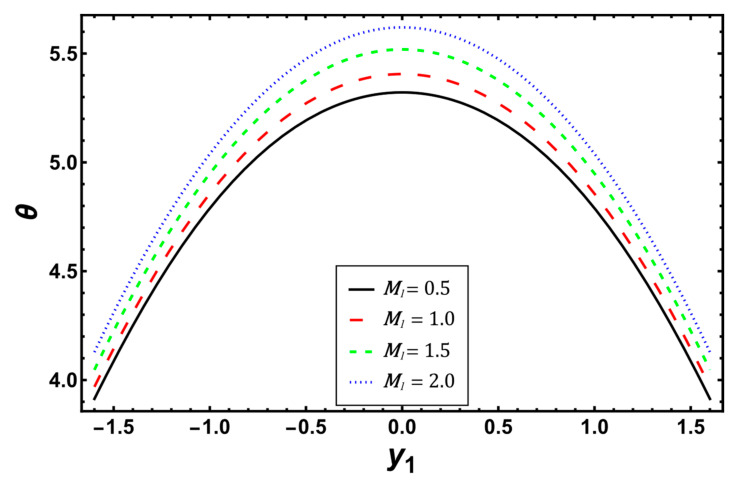
Temperature profile by varying “$M_{1}$” when a=0, x=0, η=0.8, $Bi=0.5,\;\phi_{M}=0.02,\;\varepsilon =2.0,\;\beta_{2}=0.1,\;\phi_{Z}=0.03,\;Br=0.3,\;Nr=1.0,\;Gr=2.0.$

**Figure 16:**
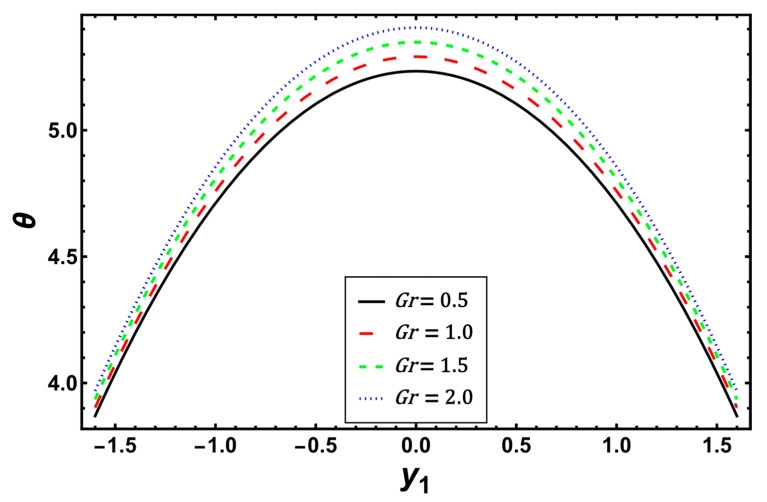
Temperature profile by varying “Gr” while a=0, x=0, η=0.8,$Bi=0.5,\;\phi_{M}=0.02,\;\varepsilon =2.0,\;\beta_{2}=0.1,\;M_{1}=1.0,\;\phi_{Z}=0.03,\;Br=0.3,\;Nr=1.0.$

**Figure 17:**
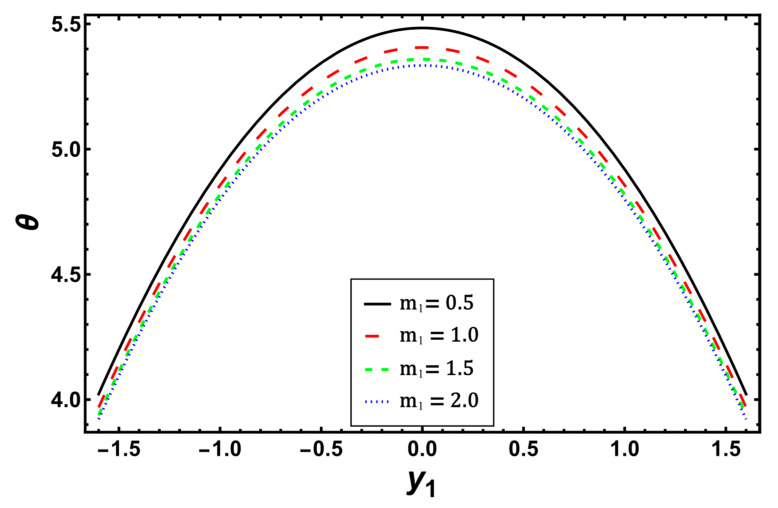
Temperature profile by varying “$m_{1}$” while a=0, x=0, η=0.8,$Bi=0.5,\;\phi_{M}=0.02,\;\varepsilon =2.0,\;\beta_{2}=0.1,\;M_{1}=1.0,\;\phi_{Z}=0.03,\;Br=0.3,\;Nr=1.0,\;Gr=2.0.$.

**Figure 18:**
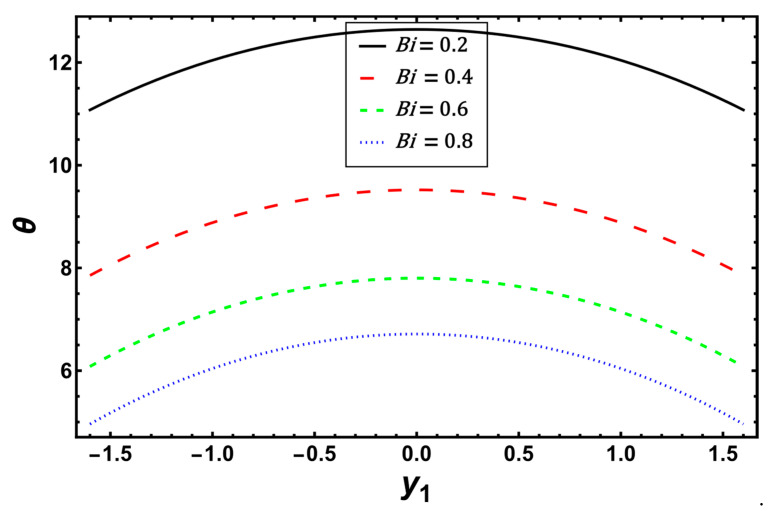
Temperature profile by varying “Bi” while a=0, x=0, η=0.8, $Bi=0.5,\;\phi_{M}=0.02,\;\varepsilon =2.0,\;\beta_{2}=0.1,\;M_{1}=1.0,\;\phi_{Z}=0.03,\;Br=0.3,\;Nr=1.0.$

**Figure 19:**
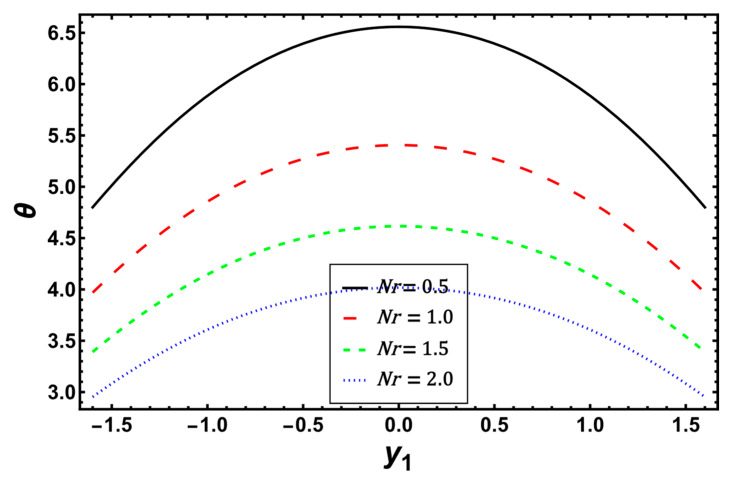
Temperature profile by varying “Nr” while a=0, x=0, η=0.8, $Bi=0.5,\;\phi_{M}=0.02,\;\varepsilon =2.0,\;\beta_{2}=0.1,\;M_{1}=1.0,\;Br=0.3,\;Nr=1.0,\;Gr=2.0.$

Figure 12, Figure 13, Figure 14, Figure 15, Figure 16, Figure 17, Figure 18 and Figure 19 relate to the impact of the Brinkman number (Br), the temperature-dependent thermal conductivity parameter (α), the Hartman number $\left(M_{1}\right)$, the Grashof number (Gr), the heat source/sink parameter (ε), the Hall parameter $\left(m_{1}\right)$, the Biot number (Bi), the thermal radiation parameter (Nr), MWCNT nanoparticle volume friction $\left(\phi_{M}\right)$, and ZnO nanoparticle volume friction $\left(\phi_{Z}\right)$ on temperature distribution. Figure 12 shows that an increase in the magnitude of temperature distribution is observed for higher values of the Brinkman number. Physically, this can be attributed to the nanofluid being heated up due to friction in the fluid layers, resulting in an improvement in the fluid temperature as the Brinkman number increases. Moreover, this is because a higher viscosity tends to suppress turbulence and promote thermal mixing, which leads to a more efficient transfer of heat between different regions of the fluid. Thus, an enhancement in the Brinkman number generally results in a higher temperature profile in the fluid. Similarly, an increasing behavior in the magnitude of temperature distribution is noted for the higher heat source parameter in Figure 13. Increasing the thermal conductivity of a fluid can enhance its ability to dissipate or absorb heat, leading to a decrease in the temperature of the hybrid nanofluid above the boundary for higher values of the temperature-dependent thermal conductivity parameter, as depicted in Figure 14. Figure 15 indicates that the temperature profile improves with an improvement in the Hartmann number, mainly due to Ohmic heating. This means that the fluid becomes hotter as the Hartmann number increases. This behavior can be explained by the fact that the magnetic field induces an electric current in the fluid, which leads to Ohmic heating. As the Hartmann number increases, the strength of the magnetic field increases, which in turn increases the electric current and the amount of heat generated by Ohmic heating. This leads to a higher temperature profile in the fluid.

**Figure 20:**
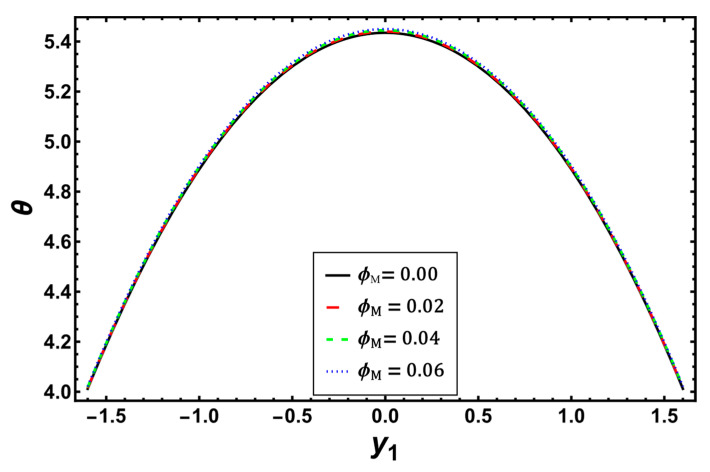
Temperature profile by varying “$\phi_{M}$” when a=0, x=0, α=0.03, $Bi=0.5,\;\varepsilon =2.0,\;\beta_{2}=0.1,\;M_{1}=1.0,\;\phi_{Z}=0.03,\;Br=0.3,\;Nr=1.0,\;Gr=2.0.$

**Figure 21:**
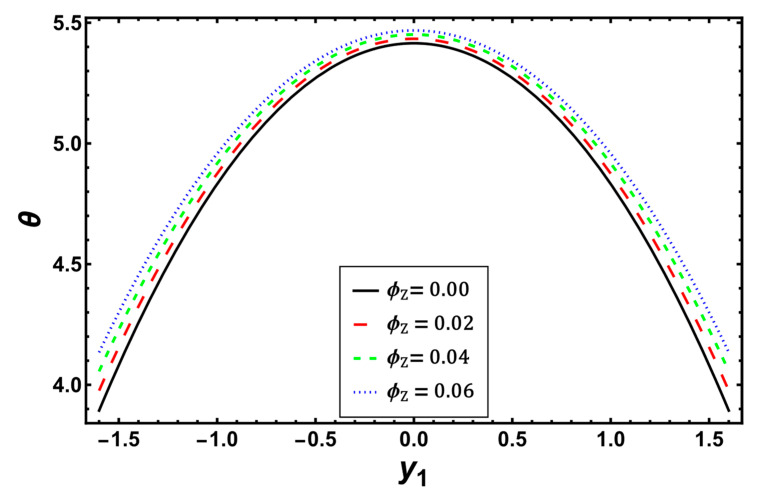
Temperature profile by varying “$\phi_{Z}$” when a=0, α=0.03, $Bi=0.5,\;\phi_{M}=0.02,\;\varepsilon =2.0,\;\beta_{2}=0.1,\;M_{1}=1.0,\;Br=0.3,\;Nr=1.0,\;Gr=2.0.$

The impact of the Grashof number on the temperature variation of the hybrid nanofluids is demonstrated in Figure 16, indicating that an increase in Gr results in an increase in fluid temperature. Figure 17 illustrates the effect of the Hall parameter on temperature distribution, showing that higher values of the Hall parameter lead to a significant reduction in the temperature of the hybrid nanofluid. This is attributed to the presence of greater Hall currents, which lessens the influence of the bulk magnetic force and subsequently decreases the temperature distribution. Furthermore, Figure 18 demonstrates the influence of the Biot number on fluid temperature and reveals a decreasing trend. The observed behavior can be attributed to the heat transfer process occurring between the fluid and the solid surface. During flow over a solid surface, heat transfer takes place from the solid to the fluid as a result of the temperature difference between them. The rate of heat transfer is determined by the Biot number, which represents the relative contribution of internal and external thermal resistance. A large Biot number indicates that the internal thermal resistance of the solid is significantly higher than the external thermal resistance, resulting in a limited heat transfer rate primarily determined by the internal resistance of the solid. This, in turn, causes the fluid temperature to remain relatively constant or decrease. Figure 19 shows that the fluid temperature drops for higher values of thermal radiation parameter Nr. Figure 20 and Figure 21 demonstrate that raising the values of $\phi_{M}$ and $\phi_{Z}$ leads to an increase in the temperature profile.

### 6.3. Entropy Generation

**Figure 22:**
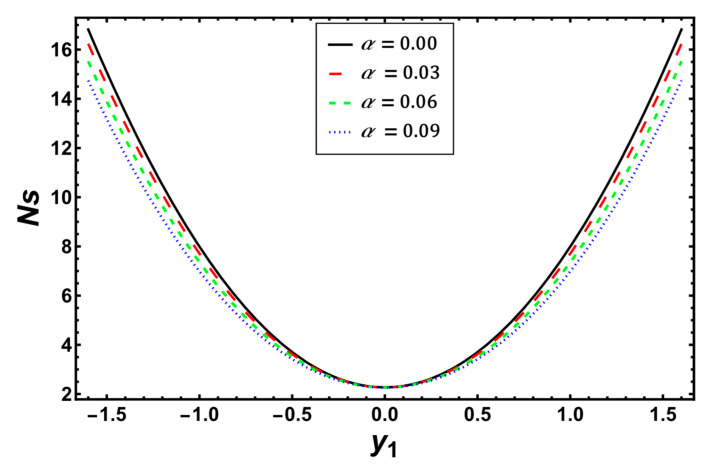
Entropy profile by varying “α” while η=0.8, α=0.03, $Bi=0.5,\;\phi_{M}=0.02,\;\varepsilon =2.0,\;\beta_{2}=0.1,\;M_{1}=1.0,\;Nr=1.0,\;Gr=2.0$.

**Figure 23:**
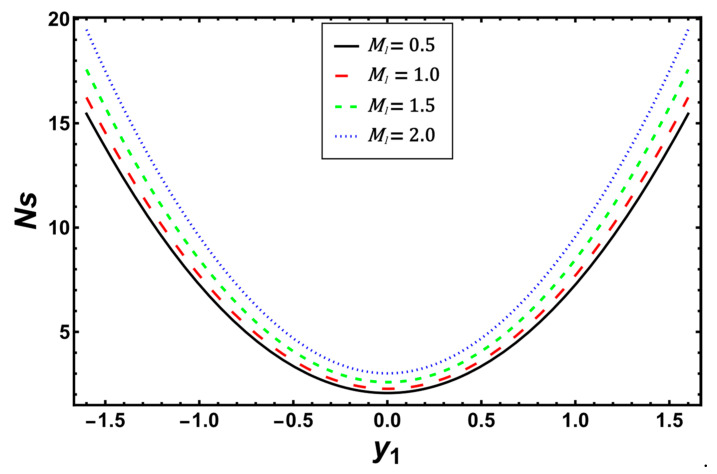
Entropy profile by varying “$M_{1}$” when a=0, x=0,$Bi=0.5,\;\phi_{M}=0.02,\;\varepsilon =2.0,\;\beta_{2}=0.1,\;M_{1}=1.0,\;\phi_{Z}=0.03,\;Br=0.3,\;Nr=1.0$.

**Figure 24:**
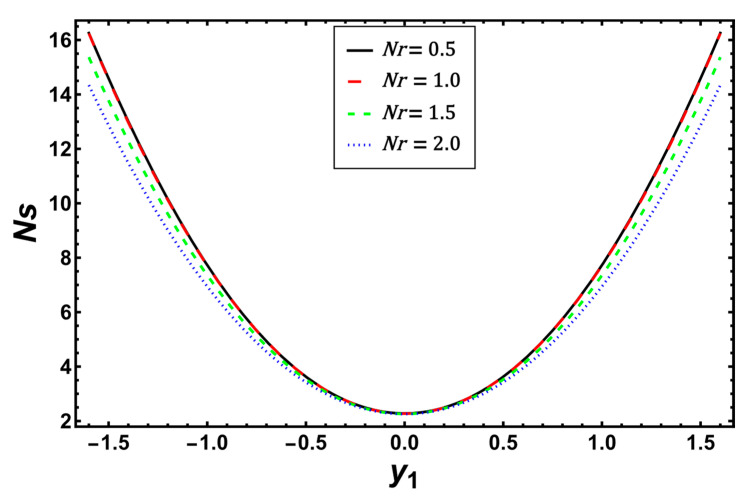
Entropy profile by varying “Nr” while η=0.8, $Bi=0.5,\;\phi_{M}=0.02,\;\varepsilon =2.0,\;\beta_{2}=0.1,\;M_{1}=1.0,\;Br=0.3,\;m_{1}=1.0,\;Gr=2.0.$

**Figure 25:**
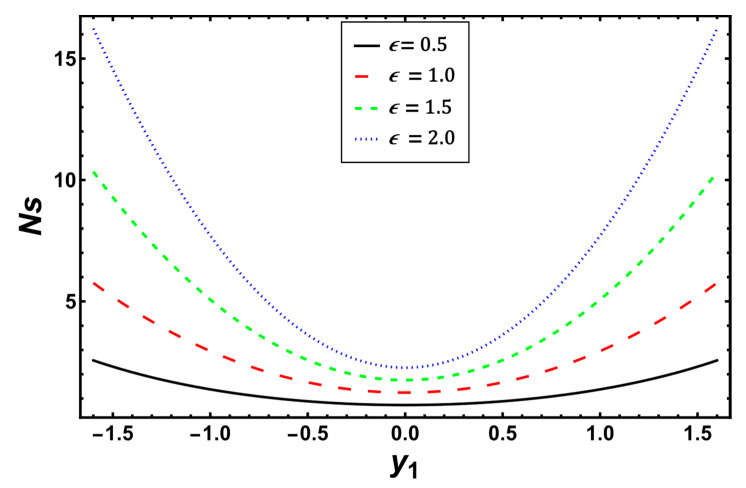
Entropy profile by varying “ε” while a=0, x=0, $Bi=0.5,\;\phi_{M}=0.02,\;Nr=1.0,\;\beta_{2}=0.1,\;M_{1}=1.0,\;\phi_{Z}=0.03,\;Br=0.3.$

**Figure 26:**
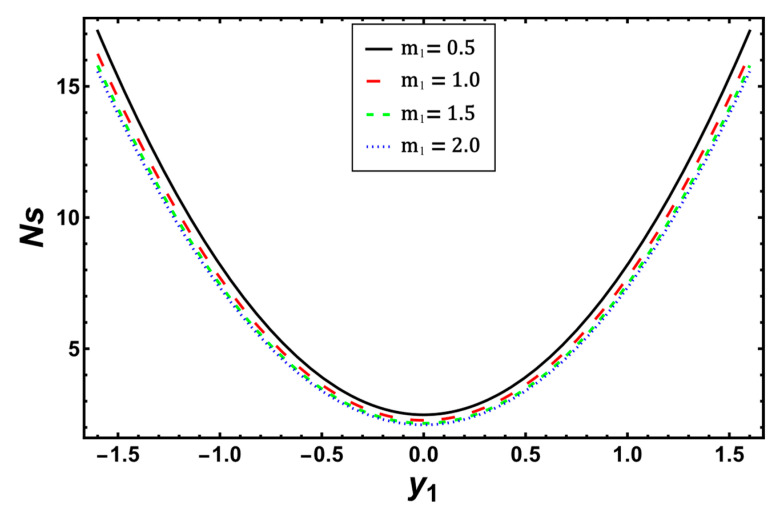
Entropy profile by varying “$m_{1}$” when a=0, η=0.8, $Bi=0.5,\;\phi_{M}=0.02,\;\beta_{2}=0.1,\;M_{1}=1.0,\;\phi_{Z}=0.03,\;Nr=1.0,\;Gr=2.0.$

Figure 22, Figure 23, Figure 24, Figure 25 and Figure 26 demonstrate how various flow parameters affect entropy generation. Evidently, the entropy generation distribution decreases near the channel walls by increasing the thermal conductivity parameter and shows a lowest value in the middle of the channel, as observed in Figure 22. This is because the increased thermal conductivity allows more efficient transfer of heat away from the fluid near the walls, reducing the quantity of entropy generated in that region. Additionally, the minimum value of entropy in the middle of the channel is due to factors such as lower fluid velocity or temperature in that region, or a more efficient transfer of heat away from the fluid due to the geometry of the channel. Figure 23 describes the change in Hartman number on the entropy profile. It is observed from Figure 22, Figure 23, Figure 24, Figure 25 and Figure 26 that the entropy distribution of hybrid nanofluid significantly increases with higher values of “$M_{1}$”. This phenomenon can be explained by the fact that when Joule heating is present, the temperature of the hybrid nanofluid increases, leading to Ohmic heating. As a result, the nanofluid generates more heat due to the effect of Joule heating, leading to a higher value of the entropy generation number. From Figure 24, it can be observed that entropy distribution lessens near the upper and lower walls by improving the thermal radiation parameter. As the thermal radiation is the transfer of heat by electromagnetic radiation, which occurs due to the temperature difference between two surfaces, therefore, by improving the thermal radiation parameter, it is expected that the heat transfer between the fluid and the walls of the channel becomes more efficient, leading to a decrease in the amount of entropy produced in those regions. In Figure 25, there is an upturn in the entropy generation profile for improving the value of the heat source/sink parameter. This means that whenever more heat is added to the system, the temperature of the system may increase, leading to higher entropy generation due to increased thermal gradients. Additionally, the increased rate of heat addition or removal may lead to more turbulent fluid flow, which could also increase entropy generation.

The entropy generation decreases with the rising Hall parameter in the channel walls, as shown in Figure 26. This can be attributed to the fact that the stronger Hall currents reduce the strength of the bulk magnetic force, resulting in a decrease in entropy generation.

### 6.4. Heat Transfer Rate

**Figure 27:**
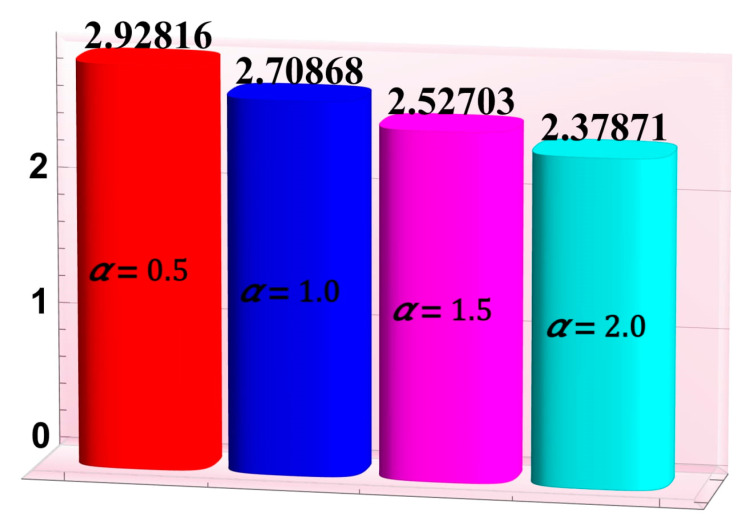
Heat transfer rates by varying “α” while a=0, x=0, η=0.8, $Bi=0.5,\;\phi_{M}=0.02,\;m_{1}=1.0,\;\beta_{2}=0.1,\;M_{1}=1.0,\;Br=0.3,\;Nr=1.0,\;Gr=2.0.$

**Figure 28:**
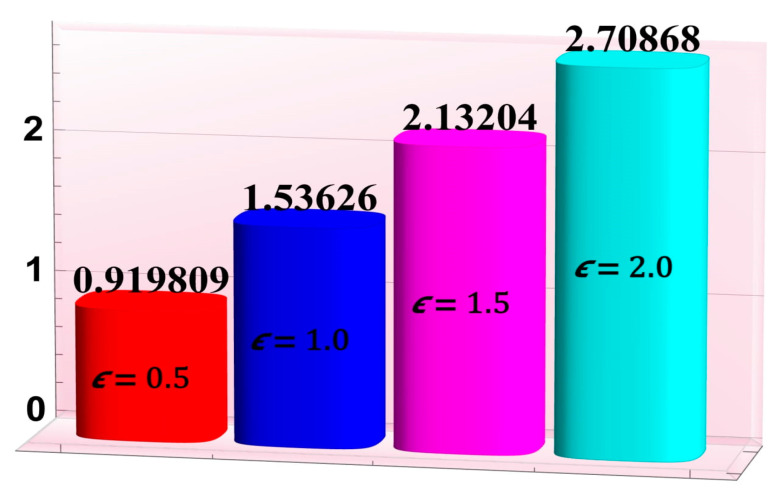
Heat transfer rates by varying “ε” while a=0, η=0.8, α=0.03, $Bi=0.5,\;\phi_{M}=0.02,\;\varepsilon =2.0,\;\beta_{2}=0.1,\;M_{1}=1.0,\;\phi_{Z}=0.03,\;Br=0.3.$

**Figure 29:**
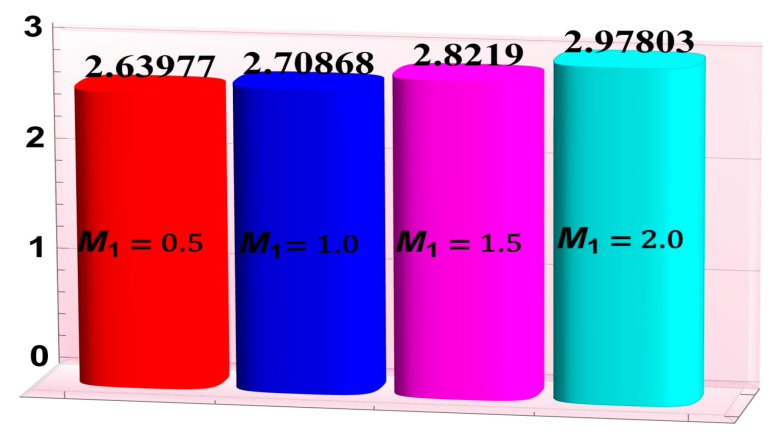
Heat transfer rates by varying “$M_{1}$” while a=0, x=0, η=0.8,$Bi=0.5,\;\phi_{M}=0.02,\;\varepsilon =2.0,\;\beta_{2}=0.1,\;\phi_{Z}=0.03,\;Br=0.3,\;Nr=1.0,\;Gr=2.0.$

**Figure 30:**
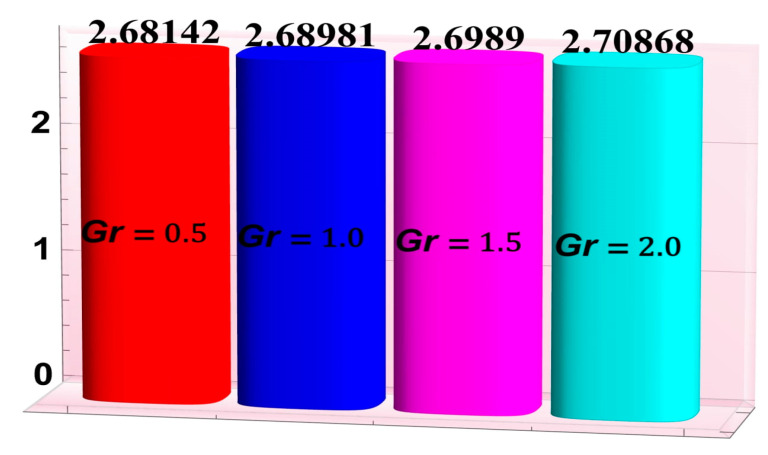
Heat transfer rates by varying “Gr” while η=0.8, α=0.03, $\varepsilon =2.0,\;\beta_{2}=0.1,\;M_{1}=1.0,\;\phi_{Z}=0.03,\;Br=0.3,\;Nr=1.0.$

**Figure 31:**
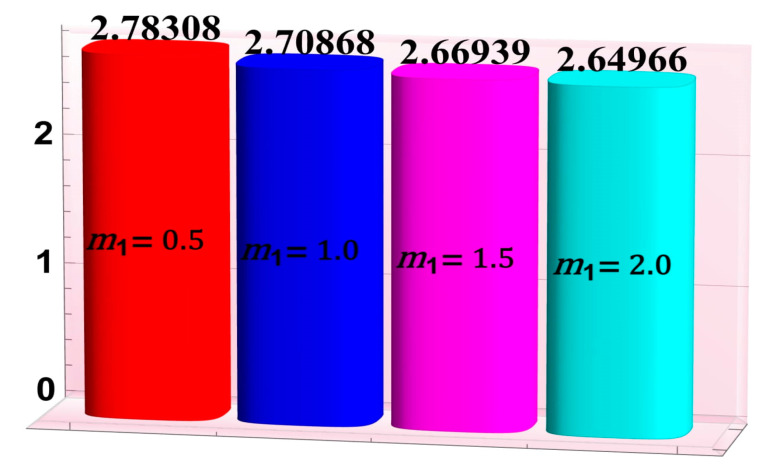
Heat transfer rates by varying “$m_{1}$” while a=0, x=0, η=0.8, α=0.03, $Bi=0.5,\;\phi_{M}=0.02,\;\varepsilon =2.0,\;\beta_{2}=0.1,\;Br=0.3,\;Nr=1.0,\;Gr=2.0.$

**Figure 32:**
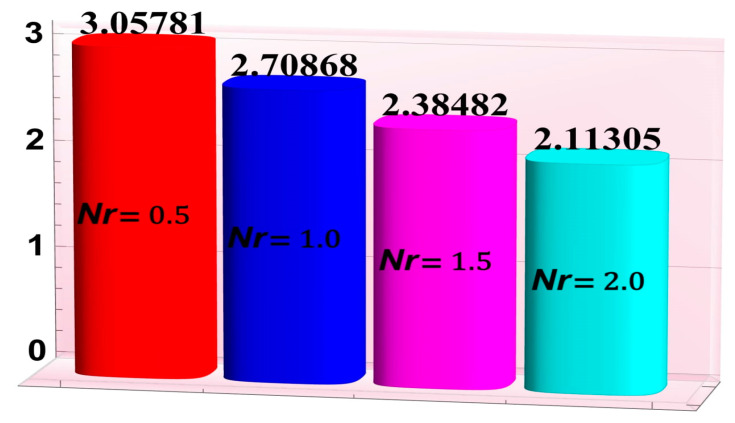
Heat transfer rates by varying “Nr” while a=0, x=0, η=0.8, $Bi=0.5,\;\phi_{M}=0.02,\;\varepsilon =2.0,\;\beta_{2}=0.1,\;M_{1}=1.0,\;\phi_{Z}=0.03,\;Br=0.3.$

**Figure 33:**
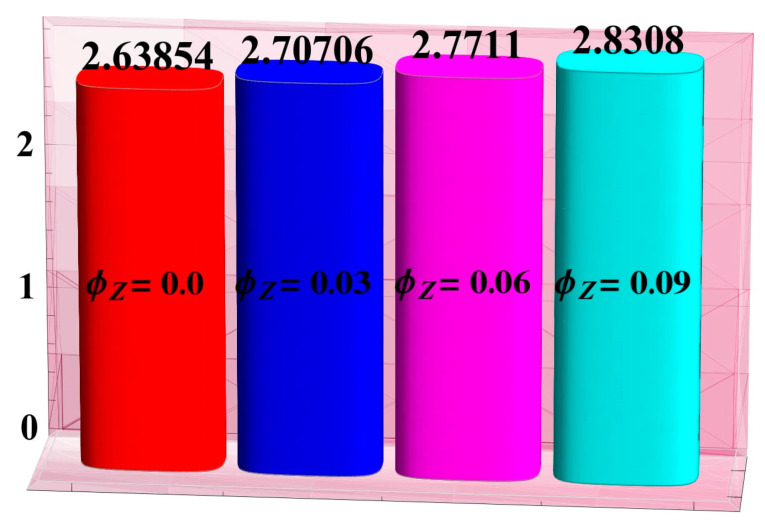
Heat transfer rates by varying “$\phi_{Z}$” while a=0, x=0, η=0.8, α=0.03,$Bi=0.5,\;\phi_{M}=0.02,\;\varepsilon =2.0,\;\beta_{2}=0.1,\;Br=0.3,\;Nr=1.0,\;Gr=2.0.$

**Figure 34:**
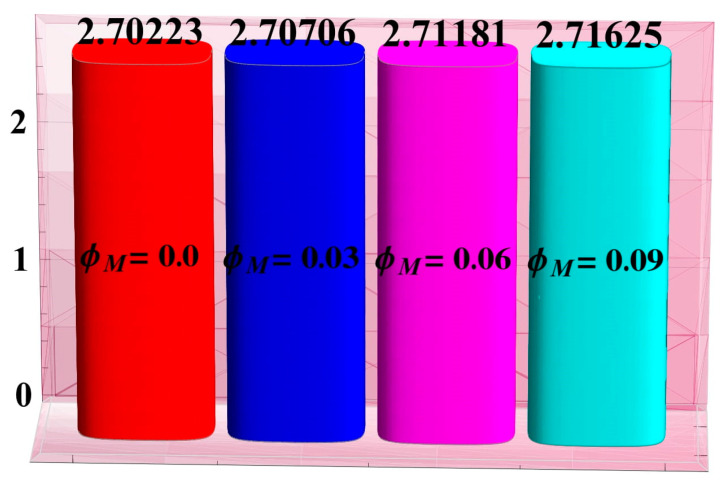
Heat transfer rates by varying “$\phi_{M}$” while a=0, x=0, η=0.8, $Bi=0.5,\;\phi_{z}=0.03,\;\varepsilon =2.0,\;\beta_{2}=0.1,\;M_{1}=1.0,\;\phi_{Z}=0.03,\;Br=0.3.$

Figure 27, Figure 28, Figure 29, Figure 30, Figure 31, Figure 32, Figure 33 and Figure 34 are presented to investigate the phenomena of the temperature-dependent thermal conductivity parameter (α), the Grashof number (Gr), the heat sink/source parameter (ε), the Hartmann number $\left(M_{1}\right)$, the thermal radiation parameter (Nr), and the Hall parameter $\left(m_{1}\right)$. Notably, the heat transfer rate at the wall for the present study is θ’(h); i.e., the derivative of the temperature at the boundary and its numerical values are given in Figure 27, Figure 28, Figure 29, Figure 30, Figure 31, Figure 32, Figure 33 and Figure 34. Figure 27 shows that increasing “α” results in a decrease in heat transfer rates at the boundary. Figure 28 and Figure 29 demonstrate the heat transfer values at the wall by varying the heat source parameter “ε” and the Hartmann number “$M_{1}$”, respectively, and indicate that increasing these parameters improves the rates of heat transfer. Figure 30 shows that increasing the Grashof number results in an increase in heat transfer rates. This reason can be clarified from the mechanism of heat transfer in the fluid flow. When a fluid is heated from below, it becomes less dense and rises due to buoyancy forces. As it rises, it carries heat with it, which is then transferred to the surrounding fluid. This process is known as natural convection. The effects of variations in the Hall and thermal radiation parameters on the heat transfer rates at the wall are depicted in Figure 31 and Figure 32, respectively, and show that the rates of heat transfer decrease for higher values of “$m_{1}$” and “Nr”. The behavior of fluid flow can be influenced by magnetic fields and thermal radiation. When a magnetic field is present, the motion of charged particles in the fluid is altered, causing a drop in the velocity of the fluid flow. Consequently, this slower movement leads to a reduction in the rate of heat transfer.

In Figure 33 and Figure 34, it can be seen that rates of heat transfer rise through an increment in hybrid nanoparticles $\left(\phi_{M},\phi_{z}\right)$. As the volume fraction of the nanomaterials in the fluid increases, the number of conductive particles in the nanofluid also increases, resulting in an increase in its thermal conductivity. This makes it easier for heat to dissipate throughout the nanofluid, resulting in a drop in the overall heat transfer rate in the wall. The rate at which heat is transferred between a wall and a nanofluid is dependent on the thermal conductivity of the fluid. An increase in the thermal conductivity of the nanofluid causes a decrease in the temperature gradient between the wall and the fluid. This reduction in the temperature gradient leads to a lower rate of heat transfer between the wall and the fluid. As a result, the heat is distributed more uniformly throughout the nanofluid, which can cause a reduction in the rate of heat transfer at the wall.

It is worth stating that the HAM solution obtained in this paper has been validated through the demonstration of convergence and qualitative agreement with previously reported results by Hayat et al. [1], Abbasi et al. [2], and Rafiq et al. [4]. The convergence of the solution was achieved by refining the auxiliary parameters until the solution is stable and consistent. Additionally, the qualitative agreement of results with previously reported ones provides further validation of the obtained solution. While quantitative comparisons between numerical and experimental or analytical data are often used to validate fluid flow simulations, the qualitative agreement observed in this study provides a strong indication of the accuracy of the solution.

## 7. Conclusions

This research investigated the impact of thermal radiation on the peristaltic motion of magnetohydrodynamic hybrid nanofluids in a symmetric channel using engine oil as the base fluid and multi-walled carbon nanotubes mixed with zinc oxide as nanoparticles. The study focused on analyzing entropy generation in particular. The main findings are as follows:

- The Brinkman number has a positive impact on temperature profile, indicating that increasing the viscosity of the fluid improves the heat transfer process. However, higher Hall parameters result in lower temperature profiles, which means that the existence of magnetic fields reduces the heat transfer efficiency.
- Enhancing the thermal radiation parameter reduces the temperature profile, suggesting that the radiation has a cooling effect on the system.
- A higher Biot number increases the magnitude of the temperature profile, indicating higher heat transfer in cases where the thermal resistance at the fluid-solid interface is small.
- Increasing the thermal conductivity parameter reduces entropy generation and the temperature profile, which implies that a more conductive fluid leads to more efficient heat transfer and lower thermal losses.
- The Hall parameter has a negative impact on entropy generation, suggesting that magnetic fields can reduce the dissipation of energy in the system. Conversely, higher Hartmann numbers lead to increased entropy generation.
- Heat transfer rates decrease with higher Hall parameters and improve with higher Grashof numbers, indicating that magnetic fields can hinder the heat transfer process while buoyancy forces enhance it.
- The heat transfer rates at the wall increase with higher heat source parameters and Hartmann numbers, indicating that the presence of magnetic fields can enhance the convective heat transfer process. However, thermal radiation has a negative effect on wall heat transfer rates.
- Higher values of Grashof and Hall parameters lead to an increase in the axial velocity in the middle of the walls, indicating that buoyancy and magnetic forces can induce fluid flow.
- The middle channel velocity profile exhibits a decreasing trend as the velocity slip parameter increases, which suggests that the slip at the fluid-solid interface can hinder the flow of the fluid.

## Figures and Tables

**Table 1:** Base fluid and nanoparticle parametric values [9,11,12,13].

Fluid/Nanoparticle	*ρ*	*ρ*	*C*	*σ*	*Β*
MWCNT	1600	3000	79	1.0 × 10^7^	44 × 10^−5^
ZnO	5600	13	495.2	7.261 × 10^−5^	4.31 × 10^−6^
Engine Oil	884	0.144	1910	700	70 × 10^−5^

## Data Availability

No new data was created or analyzed in this study. Data sharing is not applicable to this article.
